# Population-scale hand tremor analysis via anonymized mouse cursor signals

**DOI:** 10.1038/s41746-019-0171-4

**Published:** 2019-09-24

**Authors:** Ryen W. White, Eric Horvitz

**Affiliations:** Microsoft Research, Redmond, WA USA

**Keywords:** Neurology, Computer science

## Abstract

Tremors are a common movement disorder with a spectrum of benign and pathological causes, including neurodegenerative disease, alcohol withdrawal, and physical overexertion. Studies of tremors in clinical practice are limited in size and scope and depend on explicit tracking of tremor characteristics by clinicians. Data drawn from small numbers of patients observed in short-duration sessions pose challenges for understanding the nature and distribution of tremors over a large population. Methods are presented to estimate hand tremors based on anonymized computer mouse cursor movement data collected from millions of users of a web search engine. To determine the feasibility of using this signal for the estimation of the prevalence of tremors over a large population, the characteristics of tremor-like movements are computed and compared against user data that can be interpreted as self-reports, the findings of published clinical studies, and a target selection study where participants self-report hand tremors and known causes. The results demonstrate significant alignment between estimated tremors and both self-reports and clinical findings. Those with cursor tremor events are more likely to report tremor-related search interests. Variations in cursor tremor quantity and cursor tremor frequency with demographics mirror those from clinical studies. Distributions of cursor tremor frequencies vary as expected for different medical conditions. Overall, the study finds evidence for the validity of harnessing anonymized mouse cursor motion as a population-scale tremor sensor for epidemiologic studies. Feasible future applications include opt-in services for screening and for monitoring the progression of illness.

## Introduction

Tremors are involuntary, rhythmic oscillations in one or more body parts. They are the most common movement disorder found in clinical practice.^1^ Prevalence varies depending on tremor type. Essential tremor (ET) is the most common form, with prevalence rates ranging from 0.4% to 5.6%.^2^ Hand tremors are suggestive of many medical conditions, as well as other factors such as fear or fatigue.

This study focuses on resting tremors. Resting tremors occur in the absence of voluntary muscle activity. These tremors occur when a body part is relaxed and completely supported against gravity^3^ and can be exacerbated by increased cognitive load.^4^ Resting tremors are more easily identifiable in mouse cursor movements than action tremors. The low resolution of the cursor position sampling makes it difficult to detect action tremors while the cursor is in motion. Resting tremor is often symptomatic of specific medical conditions, including Parkinson’s disease (PD)^5–7^ and forms of ET.^3,4^ Dopaminergic deficiency in PD results in symptoms such as tremors and cognitive decline,^7^ evidence of which may be apparent in how people interact with computers.^8^ Prior studies have examined the effect of neurodegenerative disorders such as PD on computer mouse cursor control^9^ and cursor movement reaction time^10^ and the utility of assistive technologies to help those with motor impairments to better use computer input devices^11^ and filter tremulous movement from mouse cursors before it reaches computer interfaces.^12^ Others have used smartphone accelerometers to assess tremor frequencies.^13^ These studies are often small scale, limiting their generalizability, and require explicit measurement of tremor characteristics, which is both burdensome and unnatural. In contrast, log-based monitoring requires no additional action from patients or clinicians and can be deployed longitudinally at massive scale.^14,15^ Prior studies of the logs from millions of search engine and social media users have highlighted opportunities in using these data for the early detection of medical conditions, such as pancreatic cancer^16^ and lung cancer,^17^ and disease surveillance in general.^18,19^

The availability of large-scale, longitudinal computer mouse cursor movement data presents an opportunity to study hand tremors in ways that are infeasible in limited-duration laboratory settings or brief bedside evaluations. Numerous studies of the epidemiology of tremors can be enabled using anonymized population-scale data, including cohort analyses based on factors such as geographic location and demographics. With explicit consent, user-centric screening services housed within search engines could be employed to inform at-risk individuals and to perform longitudinal studies to track the status and progression of known illness.

Mouse cursor motion data have been used in prior studies and applications, including measuring search engine relevance,^14^ prefetching search results,^20^ detecting click and identity fraud,^21,22^ tracking attention in computer users,^23,24^ computer interface design,^25^ measuring website engagement,^26^ and searcher intent understanding.^27^ The timing of keystrokes in search queries has also been used for population-scale physiological sensing.^28^ Most relevant to this study is prior research on applying cursor motion data to detect the possibility of neurological disease^8^ or anxiety^29^ in search engine users.

This study continues and significantly expands on earlier work,^8^ which focused on discriminating between PD and ET with a machine-learned classifier that employed >50 search-log features, most of which were non-cursor based and only two features that were based on cursor tremor estimates. In ref. ^8^, little detail is provided about the methodology used for tremor estimation and no attempt is made to validate tremors detected from search logs, two shortcomings that are addressed in this article. Validation of the presence of tremors is essential in establishing the generalizability of the tremor estimation methods beyond a single application. We focus here on cursor-based tremor estimation. We present a detailed description of the log-based tremor estimation methodology and report on four complementary and comprehensive validation analyses, including a 1000-person target selection study independent from the search logs which shows that large-scale tremor estimation is feasible. We consider the feasibility of discriminating between PD and ET based on known differences in tremor frequency distributions in these diseases.^30^ The validation of the tremor estimation methods provide evidence that anonymized cursor motion can be harnessed as a population-scale tremor sensor, with applications including epidemiologic studies and opt-in services for screening and for monitoring disease progression in individuals.

We collect cursor logs from an anonymized sample of millions of searchers on the Microsoft Bing search engine (http://bing.com) and analyze the data retrospectively. Post hoc validation of cursor tremor estimates is challenging given only anonymized log data. As a second channel of information, we analyze correlations between observed cursor tremors and searchers’ self-reported interests in tremors as expressed through their search queries. Clinical knowledge about the influence of demographics and specific medical conditions on cursor tremor prevalence and characteristics is also considered. Finally, a target selection study was performed, where 1000 remote participants selected on-screen targets and self-reported hand tremors and their known causes. Analyzing the cursor movements from this study provided additional validation of the cursor-based tremor estimation methodology.

## Results

Four different analyses were performed to assess the validity of cursor-based tremor estimation: (a) connection with searchers’ interests as indicated in their search queries; (b) differences in cursor tremor prevalence and frequencies based on demographics; (c) shifts noted in the distribution of cursor tremor frequencies when conditioned on observing evidence in searchers’ queries of having received a clinical diagnosis, and (d) a target selection study where participants selected on-screen targets with their mouse pointer (and all cursor movements were recorded) and self-reported hand tremors and associated medical conditions.

### Queries as self-reporting

Individuals affected by medical symptoms and conditions are likely to search for information about them on web search engines.^31,32^ Contingency matrices were generated for whether searchers had one or more cursor tremor events (yes/no) and whether they performed one or more searches for tremors or related synonyms (e.g., “tremors,” “shaky hands,” etc.) (yes/no) over the observation period. Percentage deviations for each cell (i.e., the difference from expected, defined as (observed – expected)/expected × 100) were computed during Chi-squared analyses (where observed = frequency count in each cell and expected = (row total × column total)/grand total): +0% means that the combination is observed as expected, while +100% means it is observed twice as much as expected. Percentage deviation increases with the number of oscillations used to define a cursor tremor event (*n*_osc_) from 15% at *n*_osc_ ≥ 1 to 190% at *n*_osc_ ≥ 4 and is significant across all tested values of *n*_osc_ in the range^1,33^ (Chi-squared tests: all *χ*^2^(1) ≥ 94.76, all *p* < 0.001, all *φ* (effect sizes as phi correlations) >0.003). The searcher counts for *n*_osc_ ≥ 2 are reported in Table 1 for all tremor searches (row (a)) and for filtering tremor searchers to those who issued queries that show stronger evidence, which are referred to as “experiential queries” (row (b)). Experiential queries are typically first-person statements providing stronger evidence of tremor incidence (e.g., “my shaky hands,” “I have tremors”) than episodes of general tremor searching (excluding, using look-up tables, queries such as those that were natural language questions, related to others (e.g., friends, relatives), or related to domestic animals). Experiential queries have been leveraged in prior research as evidence of clinical diagnosis in search-log studies of brain disease,^8^ cancer,^17^ and pregnancy.^34^ These first-person statements offer more evidence that observed tremor searches are connected to personal experiences with tremors. Later, this is extended to experiential diagnostic queries: first-person statements containing evidence of clinical diagnosis. Table 1 shows that the percentage deviation for yes–yes (highlighted) is higher for experiential tremor searchers (+120%) than for all tremor searchers (+99%). Overall, the results show that searchers with cursor tremor events are more likely to self-report tremor-related interests. These findings, especially those for experiential searchers (Table 1, row (b)), offer some confirmatory evidence that cursor-based tremor estimates capture tremors experienced by searchers.

**Table 1:** Contingency tables with counts of searchers and percentage deviations from expected counts (shown in parentheses) broken down by the presence/absence of tremor-related searches and the presence/absence of cursor tremor events (all filtered to events with *n*_osc_ ≥ 2)

Searcher group	1+cursor tremor events	1+tremor search	
		Yes	No
(a) “Tremor search” means any tremor-related search	Yes	565 (+98.9%)	2,739,486 (−0.01%)
	No	292 (−49.0%)	5,525,048 (+0.005%)
(b) “Tremor search” means any experiential tremor-related search	Yes	99 (+119.6%)	2,739,952 (−0.002%)
	No	37 (−59.3%)	5,525,303 (+0.001%)

Results are reported for searchers with (a) any tremor-related searches (Chi-squared test: *χ*^2^(1) = 415.5, *p* < 0.0001, effect size as phi correlation (*φ*) = 0.007) and for (b) any experiential tremor-related searches (Chi-squared test: *χ*^2^(1) = 96.4, *p* < 0.0001, *φ* = 0.003). Searcher counts for within-searcher co-occurrence of tremor search and cursor tremor (yes–yes cells in rows (a) and (b)) are significantly above expected in both cases and higher when the positive examples of tremor searches are experiential cases (b) rather than any tremor-related search (a)

### Searcher demographics

The appearance of similar trends in mouse cursor tremor estimates would add further supportive evidence on the validity of cursor motion as a tremor sensor. In total, 2,740,051 searchers (14.3% of all 19M searchers in the dataset) had at least one cursor tremor event with *n*_osc_ ≥ 2 (7,288,536 cursor tremor events in total). The mean average (*M*) number of cursor tremor events per searcher was 2.62 (standard deviation (SD) = 4.99, median = 1). The average cursor tremor frequency per searcher was 6.81 hertz (Hz) (SD = 4.04 Hz, median = 6 Hz) and the macro-average cursor tremor frequency (averaged within the observed activity of each searcher and then again over all searchers) was 6.86 Hz (SD = 3.46 Hz, median = 6 Hz).

**Fig. 1:**
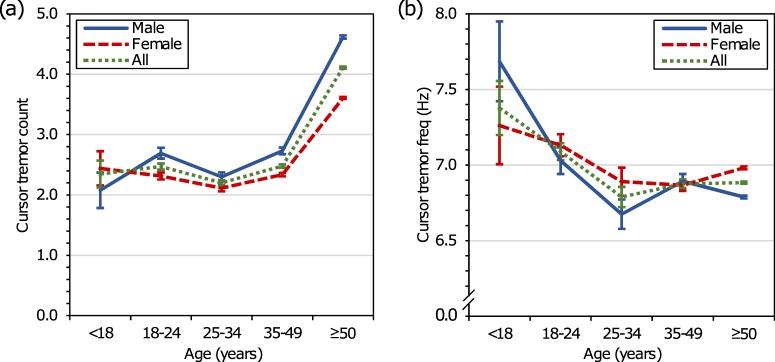
Average cursor tremor count and average cursor tremor frequency, by age and gender. **a** Mean average number of cursor tremor events per age group and gender, and **b** mean average cursor tremor frequency per age group and gender. Error bars denote standard error of the mean. To compute the macro-average used in **b**, the average cursor tremor frequency per searcher is computed and then averaged again across all searchers in each age group category. This ensures that no single searcher unduly influences overall statistics

Age and gender classifications generated by proprietary statistical classifiers from the Bing search engine were available for approximately 300k of the 2.7M searchers in the cursor tremor dataset with *n*_osc_ ≥ 2 (11.1%). The age and gender classifiers were trained by another team at Microsoft on longitudinal search queries and search-result clicks from a separate set of web searchers for whom self-reported demographics were available. The accuracy of both classifiers is approximately 80%. For each of the 300k searchers, the age classifier provided an age estimate comprising one of the five groups: <18 years (0.03% of cursor tremor searchers), 18–24 years (1.71%), 25–34 years (1.20%), 35–49 years (6.09%), and ≥50 years (90.96%). Gender classifications were divided between male (50.51% of the 300k searchers) and female (49.49%). Figure 1 summarizes cursor tremor quantity and cursor tremor frequencies by estimated age and gender. Note that statistically significant differences in the average number of queries submitted or the amount of time spent on result pages were not observed between the different age groups and between the different genders.

Two-way analyses of variance were conducted on the influence of age and gender on cursor tremor counts and cursor tremor frequency. All effects were statistically significant at the *α* = 0.05 significance level apart from gender. The main effects for age were significant for cursor tremor count (*F*(4,298636) = 234.26, *p* < 0.0005) and cursor tremor frequency (*F*(4,298636) = 45.46, *p* = 0.002), with small effect sizes (count: *η*^2^ = 0.004, frequency: *η*^2^ = 0.0007). Tukey post hoc tests indicate significant differences in cursor tremor counts between ≥50 years (*M* = 4.11, SD = 8.49) and all other groups apart from <18 years (*M* = 2.35, SD = 2.24), but only significant differences in cursor tremor frequency between ≥50 years (*M* = 6.88 Hz, SD = 3.32 Hz) and <18 years (*M* = 7.38 Hz, SD = 3.65 Hz) and 18–24 (*M* = 7.09 Hz, SD = 3.57 Hz). Although women are more likely to present with tremors in early PD,^35^ few studies have offered evidence of significant gender effects on tremor characteristics.^36^ The main effects for gender were not significant for cursor tremor count (*F*(4,298636) = 0.72, *p* = 0.396) and cursor tremor frequency (*F*(4,298636)= 0.29, *p* = 0.590). The interaction effects were significant for cursor tremor count (*F*(4,298636) = 9.46, *p* < 0.0005) and cursor tremor frequency (*F*(4,298636) = 4.07, *p* = 0.003).

### Evidence of clinical diagnosis

Clinical studies have shown that tremor frequencies vary per medical condition.^37^ Another way to validate the cursor-based tremor estimates is to examine whether the expected changes in tremor frequency for different medical conditions are also evident in cursor-based tremors. PD and ET were selected as the target conditions since resting tremors are observed in both conditions,^3,6^ the conditions are prevalent and affect millions of people worldwide, and they have distinct tremor frequency distributions. As with the earlier analysis of searchers’ self-reported tremor interests, first-person experiential diagnostic queries (containing evidence suggesting that the user was diagnosed with a medical condition) such as “just been diagnosed with parkinsons” or “treating my essential tremor” issued on the Bing search engine were used as diagnostic evidence. The patterns used to identify experiential diagnostic queries are listed in Supplementary Table 1. Cases exhibiting evidence that diagnostic queries were invalid, e.g., questions, related to others (e.g., friends, relatives), related to domestic animals, and so on, were excluded. Only cursor tremor searchers who issued one or more valid experiential diagnostic queries for PD or ET were included in this part of the analysis.

As may be expected, experiential diagnostic queries occur infrequently in search log data. There were few searchers in the 17-day experiment from mid-September until early October who issued experiential diagnostic queries for PD or ET. To find more experiential diagnostic searchers in the cursor tremors dataset for each condition, the observation period to identify experiential diagnostic PD or experiential diagnostic ET cursor tremor searchers was extended back 6 months before the start of the experiment to March 2017. The extended time frame was only used to find additional experiential diagnostic searchers; all analysis of cursor tremor events was still only performed on data from the 17-day observation period. Any experiential diagnostic search from a cursor tremor searcher appearing in that extended time was counted as evidence of clinical diagnosis. In total, 157 experiential diagnostic PD cursor tremor searchers and 109 experiential diagnostic ET cursor tremor searchers were discovered in the logs, contributing 754 and 519 cursor tremor events at *n*_osc_ ≥ 2, respectively. The cursor tremor frequency distributions for searchers in these two groups were computed and compared against the cursor tremor frequency distribution across all searchers.

**Fig. 2:**
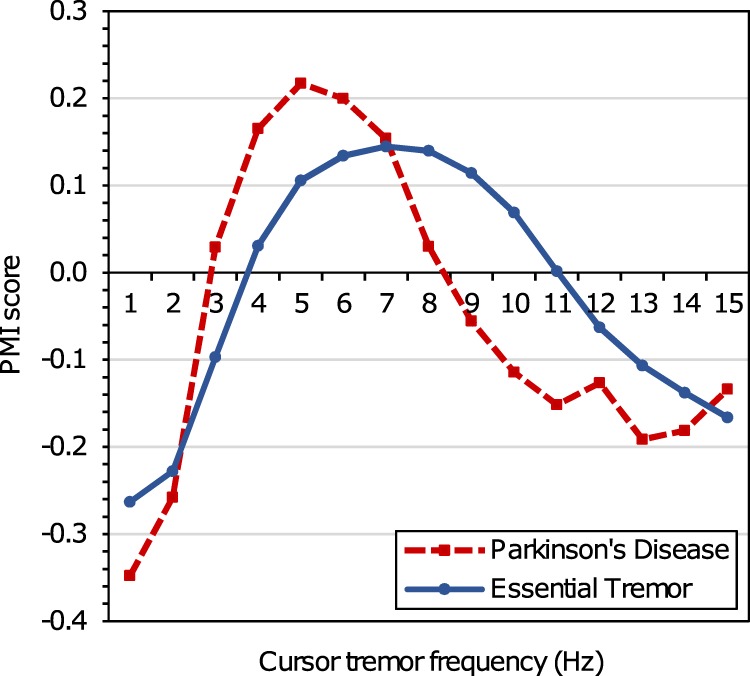
Pointwise mutual information (PMI) score by cursor tremor frequency, by medical condition. PMI score represents the change in probability of observing a specific cursor tremor frequency given evidence of condition diagnosis over the background distribution, computed across all cursor tremor events independent of condition. The PMI score distributions per cursor tremor frequency are shown for experiential diagnostic Parkinson’s disease searchers (dashed line) or experiential diagnostic essential tremor searchers (solid line)

Pointwise mutual information (PMI)^6,38^ is a measure of association from information theory and statistics. PMI was used to quantify the change in probability of observing a specific cursor tremor frequency given evidence of condition diagnosis over the background across all cursor tremor events, independent of the condition (i.e., log[*p*(tremor frequency|evidence of condition)/*p*(tremor frequency)]). The PMI score is computed at each frequency in the range [1 Hz, 15 Hz]. Figure 2 shows the PMI score distributions for PD and ET. For each condition, the regions where the cursor tremor frequency values are higher than expected (PMI > 0) correspond to tremor frequency ranges identified during clinical studies: Parkinsonian tremors (3–7 Hz)^30,39,40^ and ETs (4–12 Hz).^41^

For ET, the region of PMI > 0 (4–10 Hz) has a lower upper threshold than the 4–12 Hz reported in clinical studies.^41^ Explanations for this include a right skew in the age distribution of searchers in the cursor tremor dataset: >90% of cursor tremor searchers are aged ≥50 years and tremor frequencies can decrease with age,^42^ and the nature of the task: search is cognitively demanding^43^ and tremor frequencies can be lower for more focused tasks.^44^

### Target selection

In total, there were 1000 participants in the target selection study. In all, 6.7% self-reported experiencing hand tremors and 2.6% self-reported ET as the known cause of their hand tremors (aligned with prior studies^2,45^). Participants who self-identified with no hand tremor are regarded as controls. The gender of those reporting tremors was distributed as 55.2% male (*n* = 35), 41.8% female (*n* = 28), and 6.0% (*n* = 4) did not report their gender. There were six age groups: 18–24 years (10.5%, *n* = 7), 25–34 years (41.8%, *n* = 28), 35–44 years (9.0%, *n* = 6), 45–54 years (6.0%, *n* = 4), 55–64 years (20.9%, *n* = 14), and ≥65 years (9.0%, *n* = 6). Other medical conditions were less prevalent in the self-report data (e.g., stroke [0.7% of participants], multiple sclerosis [0.5%], and PD [0.2%]). In the raw cursor data (no downsampling), the average number of cursor events per task was 1601 (SD = 1109), the average distance traveled by the mouse cursor per task was 19,735 pixels (px) (SD = 9364 px), and the average time per task was 113 s (SD = 57 s).

Cursor tremor events were observed for 73.1% of participants (*n* = 49 of 67) who self-reported hand tremors versus 3.4% of controls (*n* = 32 of 933) (*Z* = 20.23, *p* < 0.001). Cursor tremor frequencies for the 73 cursor tremor events observed from the 26 participants self-reporting ET (*M* = 9.12Hz; *SD* = 5.31Hz) significantly differed from frequencies of all other cursor tremor events in the dataset (*M* = 5.93Hz; *SD* = 4.49Hz; *t*(275) = 3.70, *p* < 0.01). The frequency range with PMI score > 0 was 6–12 Hz; this is toward the upper end of the range of tremor frequencies observed in clinical studies on ET,^41^ perhaps reflecting the younger demographic in the participant population of the target selection study.^46^

## Discussion

The findings of this feasibility study demonstrate alignment between cursor tremor events and both self-reported interest in tremors and findings of clinical studies, providing evidence in support of using cursor movements as a population-scale sensor of cursor tremor events. Clinical studies have shown that tremor incidence increases with age^47^ and that tremor frequencies decrease with age.^46,48^ The variations in cursor tremor quantity and cursor tremor frequency with searcher demographics observed in this study are concordant with findings in those studies, providing additional supportive evidence for mouse cursor motion as a means of tremor estimation. The alignment between cursor tremor distributions from searchers issuing experiential diagnostic queries and tremor distributions from clinical studies^30,39–41^ further supports the use of mouse cursor motion for tremor estimation. The consistency offers additional confirmatory evidence that observed cursor tremor events capture real tremors and are less likely to be attributable to other possible causes, such as myoclonus.^49^ While the results are promising, additional validation is required. The sensitivity and specificity of detecting tremor via the use of computer mice needs to be determined with patients and controls. Such studies will help answer important questions such as the level of severity of cursor tremors required to separate them from other extraneous movements.

The data analyzed were from a short (17-day) experiment for a sample of search traffic on a specific search engine (Bing). First-person experiential queries were used to flag likely tremor sufferers and experiential diagnostic queries were used to identify those likely to have been diagnosed with medical conditions. Experiential queries have been used in previous studies to identify searchers exhibiting evidence of diagnosis,^8,17,34^ but more work is needed to validate this approach with patients, especially given that misdiagnosis of both PD and ET are common, as is confusion over general Parkinsonism.^50^ The relationship between search tasks and characteristics of tremor-like movements needs to be understood, including the presence and influences of anxiety, including anxiety induced in the process of search and retrieval (e.g., searching on alarming medical conditions^32^). The target selection study offered an additional source of validation independent of the search logs and where participants self-reported known causes of hand tremors in line with established prevalence rates.^45^ More cursor movement data are needed over a longer time frame and multiple application settings to obtain more robust insights, including how cursor tremor frequencies change for searchers over time.^51^

Future work includes exploring the application of these methods to additional tremor types and other conditions with motor symptoms, as well as studying environmental sources of vibration (e.g., computer usage on airplanes or on public transportation). There are many types of tremor, including rhythmic and non-rhythmic and task or situation-related tremors, and almost all with overlapping frequency ranges. Different tremors need to be recorded in different ways, e.g., resting tremors will disappear with intentional mouse movements. High frequency tremors, such as exaggerated physiological tremor (caused by fatigue, metabolic diseases, alcohol withdrawal, and others), may not be recordable by a computer mouse due to friction and low sampling rates. Action tremors may be detectable from cursor trails given more granular tracking of computer mouse cursor position to estimate tremor behaviors while the cursor is in motion. Factors that could affect the reliability of the cursor tremor estimation also require further investigation. Computer mice move in proportion to the velocity and/or acceleration of mouse movements.^52^ Mouse motion therefore does not provide a linear transduction of tremor amplitude, which could be biased by higher tremor frequencies. The current study focuses on small cursor movements (≤15 px), which may be less affected by these biases, and filters cursor tremor events to those with frequencies from 1 to 15 Hz to reduce errors attributable to sampling noise. A thorough assessment is required of possible bias in tremor amplitude related to the use of cursor data. Standardizing cursor tremor estimation methods across different system settings (e.g., screen resolutions, display dimensions, input device types, mouse sensitivities) may improve tremor estimates. This study could not control for these settings in the retrospective log analyses, but the target selection study did control for display dimensions and input device type.

The methods described in this article are meant to complement, not replace, traditional studies of tremor by offering a new lens on tremors across thousands or millions of individuals. Logs need to be used carefully: logged search activity can be affected by many aspects of the search experience including query autocompletion^53^ and result page content.^54^ The nature of the search query (e.g., informational or navigational) can also impact people’s cursor movements on result pages.^15^ Log-based monitoring is especially useful in studies where broad regional analyses are paramount over attention to individual detail, such as monitoring large-scale trends and epidemics, or for other applications such as population-scale physiological sensing,^28^ but they are unlikely to be sufficiently discriminative for diagnostic inferences on their own.

As mentioned earlier, log-based tremor estimates can be combined with other signals to detect evidence of neurodegenerative disease.^8^ Beyond contributing as features in automated disease detection, there are other possible applications of cursor tracking. These include studying cursor-based tremors in cohorts generated based on location, demographics, or other data (e.g., topical interests as inferred from query logs), an analysis that is challenging to do at scale using current methods. Longitudinal studies of cursor-based tremors could enable the provision of opt-in services for screening for neurological disorders and for the tracking of disease progression in people with confirmed neurodegenerative diseases. Such studies could also assist physicians by providing longitudinal patterns or trends in tremors using data collected unobtrusively from people’s everyday activities, a signal that is currently unavailable for clinical use. Technologies such as Personal KinetiGraph (http://globalkineticscorporation.com/the-pkg-system) or Kinesia (http://glneurotech.com/kinesia) have a similar objective but impose additional burden by requiring that patients use devices such as smart watches that provide continuous monitoring and assessment of tremors. Search log analyses can complement these methods on an individual basis and can uniquely provide population-scale tremor insights across millions of search engine users.

In applications, it is of paramount importance to ensure user privacy and to provide users with means to opt-in and easily opt-out of any services related to tremor estimation. Looking ahead on these and similar applications and analyses, search providers must keep their users informed and in control. Searchers must consent to long-term tracking, and search providers should clearly communicate both what data are being recorded and how they are being used. Follow-up studies are required with known tremor patients with different medical conditions and clinicians to further validate tremor estimation via mouse cursor movements and evaluate the diagnostic and prognostic utility of this promising new class of motor signal.

## Methods

### Large-scale search log study

Anonymized data were collected from >19M users of the Bing search engine in the United States (a random sample of the larger user population). Data were collected from consenting users (per the terms of the end user license agreement) as part of a separate, unrelated experiment to measure user engagement with the search engine. Data collection did not influence the presentation of search results or other aspects of the user experience. The experiment ran for 17 days, from September 19, 2017 to October 5, 2017 inclusive and covered all queries from searchers included in the study. It used existing methods for mouse cursor tracking at scale on the Bing search results page.^14,15^ Mouse cursor position was logged every 10 px of computer mouse motion. Time-based sampling and interpolation of cursor positions were not used. Ten pixels corresponds to approximately 2.5 mm in on-screen distance at the standard resolution of 96 dots per inch. Only mouse cursor movements were tracked in this study; on-screen touch or gestural movements were not monitored. The information in the logs is insufficient to differentiate between cases where searchers were manipulating a physical computer mouse controlled with a hand or using a trackpad controlled by a single finger.

All analysis was conducted offline on existing data. The data were recorded in such a way that searchers cannot be identified, directly or through identifiers linked to them. Each searcher is represented by a unique identifier (one-way hash) stored in a web browser cookie that persisted over multiple search sessions. An external review of the study protocol by Western Institutional Review Board (WIRB) approved the search log study, with the determination that the research met the requirements for IRB exemption under 45 CFR 46.101(b)(4).

Sequences of consecutive cursor location data points are used to estimate hand tremor events. Although the hand is at rest, tremors may still cause observable variations in the mouse cursor position. The simple method developed to estimate hand tremors from large-scale cursor data defined a cursor tremor event as an observed tremor-like movement with (a) at least one oscillation (i.e., a short cursor movement in one cardinal direction [north, south, east, or west] followed by an immediate movement in the opposite direction) (e.g., move the cursor north [toward the top of the screen] and then move the cursor south [toward the bottom of the screen]), and (b) cursor movements of ≤15 px in both directions. Although most cursor movements were recorded every 10 px, the observed distances were occasionally slightly larger than that due to factors including network latencies, mouse precision, computational delays, etc. The tremor estimation threshold was set to 15 px to ensure that these cursor events were still included in the analysis. Applying this restriction helps to clearly distinguish cursor-based tremors from intentional actions, such as clicking on a hyperlink or right–left movements associated with reading.^24^ It is unlikely that computer users would voluntarily produce such small oscillations in cursor position. Cursor movement patterns that did not meet these criteria were ignored. Given the coarseness of the cursor data, more sophisticated tremor detectors such as those utilizing zero crossing (e.g., ref. ^55^) could not be used.

In practice, cursor movements will rarely occur precisely along the horizontal and vertical axes between east–west and north–south, respectively. To improve the robustness of the tremor estimation and allow minor deviations in cursor direction, 90-degree regions were established around each of the four cardinal directions: 45° before the cardinal direction and 45° after the cardinal direction. For example, “north” was defined as the region from north west (315°N) to north east (45°N). The minimum number of oscillations (*n*_osc_) required for a sequence of cursor movements to be classified as a cursor tremor event can be adjusted per precision and recall requirements (higher values of *n*_osc_ improve precision but harm recall). The total oscillation time in seconds is determined using the timestamps for each log entry and used in conjunction with oscillation counts to estimate tremor frequencies in hertz (Hz). There can be multiple cursor tremor events per query impression, defined as an instance of the submission of a query, the generation and presentation of a results page by the search engine, and the interaction with that page by the searcher. In total, 38,148,131 cursor tremor events with at least one oscillation (*n*_osc_ ≥ 1) and frequencies ranging from 1 to 15 Hz inclusive were extracted from 5,726,038 searchers across all values of *n*_osc_ in the range.^1,33^ Cursor tremor with frequencies <1 Hz or >15 Hz are likely caused by sampling noise and are excluded from the dataset.

### Target selection study

To help further validate the use of cursor movements as a source of tremor signal, a crowdsourced study was designed that uses a target-selection methodology from previous tremor studies.^9^ The study also collected self-report data about hand tremors from participants. Participants completed a consent form at the start of the study. They were recruited from a micro-tasking vendor (http://clickworker.com) in the English-speaking United States market. Participants were asked to select (click) with their mouse cursor 25 targets (squares) positioned randomly in a 1024 × 768 px bounding box on the screen (all participants used a device with at least these display dimensions). Participation was limited to one task per participant and participants were paid 50¢ per task. The *x*- and *y*-coordinates of mouse movements and every mouse click (on target and off target) were recorded using JavaScript. Target width was randomly selected from {8 px, 16 px, 32 px}. To reduce learning effects, a random delay of 1–3 s inclusive was applied after each click on a target before the next target was displayed. After clicking a target, the center point of the next target was computed at a random angle and a random distance from {192 px, 384 px, 768 px}. If the next target position was outside the bounding box (even partially), the location was recomputed until the full target was visible. Participants were required to use a standard optical computer mouse rather than stylus pens or touchscreens. JavaScript was used to enforce the use of a computer mouse. However, it was unable to distinguish between mouse, trackball, and touchpad usage; for that, the study relied on participants’ adherence to task guidelines, where it was clearly stated that only optical mice should be used.

To understand the validity of the cursor tremor estimation method from the large-scale study, cursor data were downsampled to retain mouse cursor positions every 10 px of cursor movement and cursor tremor events were defined as oscillations of ≤15 px horizontally or vertically with a frequency of 1–15 Hz inclusive; the same definitions as earlier. Downsampling reduced the number of mouse cursor events by around 50%.

Immediately after the target selection task, participants completed a survey to collect demographics (age, gender), computer experience (years), the presence of hand tremors (yes/no), and the medical condition(s) that cause these tremors (if known). All questions were optional. The study protocol was reviewed and approved by the IRB at Microsoft Research.

Methods for both studies were performed in accordance with relevant guidelines and regulations.

### Reporting summary

Further information on research design is available in the Nature Research Reporting Summary linked to this article.

## Supplementary information

Supplementary Table 1

Reporting Summary

## Data Availability

Data that support the findings of this study are available from Microsoft, but restrictions apply to the availability of these data. Data are, however, available from the authors upon reasonable request and with permission of Microsoft.

## Supplementary information

**Supplementary information** accompanies the paper on the *npj Digital Medicine* website (10.1038/s41746-019-0171-4).

## Code availability

Custom code was written to process the search log data. This code was written in a Microsoft proprietary programming language (SCOPE) and is not usable outside of the company.

## References

[1] Jankovic J, Fahn S (1980). Physiologic and pathologic tremors: diagnosis, mechanism, and management. Ann. Intern. Med..

[2] Findley LJ, Koller WC (1987). Essential tremor: a review. Neurology.

[3] Leehey MA (2001). Tremor: diagnosis and treatment. Prim. Care Case Rev..

[4] Cohen O, Pullman S, Jurewicz E, Watner D, Louis ED (2003). Rest tremor in patients with essential tremor: prevalence, clinical correlates, and electrophysiologic characteristics. Arch. Neurol..

[5] Forno LS (1996). Neuropathology of Parkinson’s disease. J. Neuropath. Exp. Neurol..

[6] Gelb DJ, Oliver E, Gilman S (1999). Diagnostic criteria for Parkinson’s disease. Arch. Neurol..

[7] Jankovic J (2008). Parkinson’s disease: clinical features and diagnosis. J. Neurol. Neurosurg. Psychiatry.

[8] White RW, Doraiswamy PM, Horvitz E (2018). Detecting neurodegenerative disorders from web search signals. NPJ Digit. Med..

[9] Keates, S. & Trewin, S. Effect of age and Parkinson’s disease on cursor positioning using a mouse. In *Proc. ACM SIGACCESS Conference on Computers and Accessibility* 68–75 (ACM, 2005).

[10] Pullman SL, Watts RL, Juncos JL, Chase TN, Sanes JN (1988). Dopaminergic effects on simple and choice reaction time performance in Parkinson’s disease. Neurology.

[11] Findlater, L. et al. Enhanced area cursors: reducing fine pointing demands for people with motor impairments. In *Proc. ACM UIST Symposium on User Interface Software and Technology* 153–162 (ACM, 2010).

[12] Rocon E, Miranda JA, Pons JL (2006). TechFilter: filtering undesired tremorous movements from PC mouse cursor. Technol. Disabil..

[13] Joundi RA, Brittain JS, Jenkinson N, Green AL, Aziz T (2011). Rapid tremor frequency assessment with the iPhone accelerometer. Parkinsonism Relat. Disord..

[14] Huang, J., White, R. W. & Dumais, S. No clicks, no problem: using cursor movements to understand and improve search. In *Proc. ACM SIGCHI Conference on Human Factors in Computing Systems* 1225–1234 (ACM, 2011).

[15] Buscher, G., White, R. W., Dumais, S. & Huang, J. Large-scale analysis of individual and task differences in search result page examination strategies. In *Proc. ACM WSDM Conference on Web Search and Data Mining* 373–382 (ACM, 2012).

[16] Paparrizos J, White RW, Horvitz E (2016). Screening for pancreatic adenocarcinoma using signals from web search logs: feasibility study and results. J. Oncol. Pract..

[17] White RW, Horvitz E (2017). Evaluation of the feasibility of screening patients for early signs of lung carcinoma in web search logs. JAMA Oncol..

[18] Ginsberg J (2009). Detecting influenza epidemics using search engine query data. Nature.

[19] Brownstein JS, Freifeld CC, Madoff LC (2009). Digital disease detection: Harnessing the web for public health surveillance. N. Engl. J. Med..

[20] Diaz, F., Guo, Q. & White, R. W. Search result prefetching using cursor movement. In *Proc. ACM SIGIR Conference on Research and Development in Information Retrieval* 609–618 (ACM, 2016).

[21] Monaro M, Gamberini L, Sartori G (2017). The detection of faked identity using unexpected questions and mouse dynamics. PLoS ONE.

[22] Park, K., Pai, V. S., Lee, K. W. & Calo, S. B. Securing web service by automatic robot detection. In *Proc. USENIX Annual Technical Conference* 255–260 (USENIX Association, 2006).

[23] Navalpakkam, V. et al. Measurement and modeling of eye-mouse behavior in the presence of nonlinear page layouts. In *Proc. World Wide Web Conference* 953–964 (ACM, 2013).

[24] Rodden, K., Fu, X., Aula, A. & Spiro, I. Eye-mouse coordination patterns on web search results pages. In *Proc. ACM SIGCHI Conference on Human Factors in Computing Systems, Extended Abstracts* 2997–3002 (ACM, 2008).

[25] McCay-Peet, L., Lalmas, M. & Navalpakkam, V. On saliency, affect and focused attention. In *Proc. ACM SIGCHI Conference on Human Factors in Computing Systems* 541–550 (ACM, 2012).

[26] Arapakis, I. & Leiva, L. A. Predicting user engagement with direct displays using mouse cursor information. In *Proc. ACM SIGIR Conference on Research and Development in Information Retrieval* 599–608 (ACM, 2016).

[27] Guo, Q. & Agichtein, E. Exploring mouse movements for inferring query intent. In *Proc. ACM SIGIR Conference on Research and Development in Information Retrieval* 707–708 (ACM, 2008).

[28] Althoff, T., Horvitz, E., White, R. W. & Zeitzer, J. Harnessing the web for population-scale physiological sensing: a case study of sleep and performance. In *Proc. World Wide Web Conference* 113–122 (ACM, 2017).

[29] Youngmann, B. & Yom-Tov, E. Anxiety and information seeking: evidence from large-scale mouse tracking. In *Proc. World Wide Web Conference* 753–762 (ACM, 2018).

[30] Thenganatt MA, Louis ED (2012). Distinguishing essential tremor from Parkinson’s disease: bedside tests and laboratory evaluations. Expert Rev. Neurother..

[31] White, R. W. & Horvitz, E. Experiences with web search on medical concerns and self-diagnosis. In *Proc. AMIA Annual Symposium* 696–700 (AMIA, 2009). PMC2815378 20351943

[32] White RW, Horvitz E (2009). Cyberchondria: studies of the escalation of medical concerns in web search. ACM Trans. Inform. Syst..

[33] Bhidayasiri R (2005). Differential diagnosis of common tremor syndromes. Postgrad. Med. J..

[34] Fourney, A., White, R. W. & Horvitz, E. Exploring time-dependent concerns about pregnancy and childbirth from search logs. In *Proc. ACM SIGCHI Conference on Human Factors in Computing Systems* 737–746 (ACM, 2015).

[35] Haaxma CA (2007). Gender differences in Parkinson’s disease. J. Neurol. Neurosurg. Psychiatry.

[36] Elble RJ (2003). Characteristics of physiologic tremor in young and elderly adults. Clin. Neurophysiol..

[37] Elble, R. J. Tremor. In *Neuro-Geriatrics: A Clinical Manual* (eds Tousi, B. and Cummings, J.) 311–326 (Springer, 2017).

[38] Church KW, Hanks P (1990). Word association norms, mutual information, and lexicography. Comput. Linguist..

[39] Jankovic J, Schwartz KS, Ondo W (1999). Re-emergent tremor of Parkinson’s disease. J. Neurol. Neurosurg. Psychiatry.

[40] Lakie M, Mutch WJ (1989). Finger tremor in Parkinson’s disease. J. Neurol. Neurosurg. Psychiatry.

[41] Benito-León J, Louis ED (2006). Essential tremor: emerging views of a common disorder. Nat. Clin. Pract. Neurol..

[42] Marshall J (1962). Observations on essential tremor. J. Neurol. Neurosurg. Psychiatry.

[43] Gwizdka J (2010). Distribution of cognitive load in web search. J. Am. Soc. Inf. Sci. Technol..

[44] Elble RJ, Brilliant M, Leffler K, Higgins C (1996). Quantification of essential tremor in writing and drawing. Mov. Disord..

[45] Louis, E. D. & Ottman, R. How many people in the USA have essential tremor? Deriving a population estimate based on epidemiological data. *Tremor Other Hyperkinet. Mov*. **4**, 259 (2014). 10.7916/D8TT4P4B PMC4137360 25157323

[46] Marshall J (1961). The effect of ageing upon physiological tremor. J. Neurol. Neurosurg. Psychiatry.

[47] Van Den Eden SK (2003). Incidence of Parkinson’s disease: variation by age, gender and race/ethnicity. Am. J. Epidemiol..

[48] Elble RJ (2000). Essential tremor frequency decreases with time. Neurology.

[49] Caviness JN, Brown P (2004). Myoclonus: current concepts and recent advances. Lancet Neurol..

[50] Meara JO, Bhowmick BK, Hobson P (1999). Accuracy of diagnosis in patients with presumed Parkinson’s disease. Age Ageing.

[51] Hellwig B (2009). A longitudinal study of tremor frequencies in Parkinson’s disease and essential tremor. Clin. Neurophysiol..

[52] Casiez G, Vogel D, Balakrishnan R, Cockburn A (2008). The impact of control-display gain on user performance in pointing tasks. Hum. Comput. Interact..

[53] Mitra, B., Shokouhi, M., Radlinski, F. & Hofmann, K. On user interactions with query auto-completion. In *Proc. ACM SIGIR Conference on Research and Development in Information Retrieval* 1055–1058 (ACM, 2014).

[54] Agichtein, E., Brill, E., Dumais, S. & Ragno, R. Learning user interaction models for predicting web search result preferences. In *Proc. ACM SIGIR Conference on Research and Development in Information Retrieval* 3–10 (ACM, 2006).

[55] Timmer J, Gantert C, Deuschl G, Honerkamp J (1993). Characteristics of hand tremor time series. Biol. Cybern..

