# Supplementary material for: Population-scale hand tremor analysis via anonymized mouse cursor signals
Source: NPJ Digit Med. 2019 Sep 24;2:93. doi: 10.1038/s41746-019-0171-4 (PMC6760188; doi:10.1038/s41746-019-0171-4)
Supplement: Supplementary file 1 — Supplementary Table 1 [file 41746_2019_171_MOESM1_ESM.pdf]

Supplementary Table 1. Experiential diagnostic query patterns. Query pattern and relative position of medical condition is one of "Condition before or after" (condition appears immediately before or immediately after pattern in the query) or "Condition after" (condition appears immediately after pattern in the query). These patterns and conditions are used to automatically identify first-person experiential diagnostic queries in search logs. Queries matching these patterns are further filtered to exclude questions, those related to other individuals (e.g., friends, colleagues, relatives), those related to domestic animals, and so on.

**Pattern**	**Relative Condition Position in Query**
i was diagnosed	Condition before or after
i got diagnosed	Condition before or after
i was just diagnosed	Condition before or after
i was recently diagnosed	Condition before or after
i have been diagnosed	Condition before or after
i have just been diagnosed	Condition before or after
i have recently been diagnosed	Condition before or after
what now	Condition before or after
what next	Condition before or after
what happens now	Condition before or after
what happens next	Condition before or after
what comes now	Condition before or after
what comes next	Condition before or after
what can i expect	Condition before or after
what should i expect	Condition before or after
what can i eat	Condition before or after
what should i eat	Condition before or after
what can i do	Condition before or after
what should i do	Condition before or after
what to expect	Condition before or after
what to eat	Condition before or after
what to do	Condition before or after
what do i do	Condition before or after
how do i cope	Condition before or after
how can i cope	Condition before or after
how should i cope	Condition before or after
how will i cope	Condition before or after
what is my life expectancy	Condition before or after
how long until i die	Condition before or after
will i die	Condition before or after
how long will i live	Condition before or after
how long do i have to live	Condition before or after
how long do i have	Condition before or after

**Pattern (cont.)**	**Relative Condition Position (cont.)**
affairs in order	Condition before or after
just diagnosed	Condition after
just been diagnosed	Condition after
been diagnosed	Condition after
diagnosed with	Condition after
diagnosed as	Condition after
diagnosed with a	Condition after
recently diagnosed	Condition after
recently been diagnosed	Condition after
just been told i am	Condition after
just been told i m	Condition after
just been told i have	Condition after
just been told i have a	Condition after
been told i am	Condition after
been told i m	Condition after
been told i have	Condition after
been told i have a	Condition after
told i am	Condition after
told i m	Condition after
told i have	Condition after
told i have a	Condition after
dr says i am	Condition after
dr says i m	Condition after
dr says i have	Condition after
dr says i have a	Condition after
dr told me i am	Condition after
dr told me i m	Condition after
dr told me i have	Condition after
dr told me i have a	Condition after
dr said me i am	Condition after
dr said me i m	Condition after
dr said i have	Condition after
dr said i have a	Condition after
doctor says i am	Condition after
doctor says i m	Condition after
doctor says i have	Condition after
doctor says i have a	Condition after
doctor told me i am	Condition after
doctor told me i m	Condition after
doctor told me i have	Condition after

**Pattern (cont.)**	**Relative Condition Position (cont.)**
doctor told me i have a	Condition after
doctor said i am	Condition after
doctor said i m	Condition after
doctor said i have	Condition after
doctor said i have a	Condition after
physician says i am	Condition after
physician says i m	Condition after
physician says i have	Condition after
physician told me i m	Condition after
physician told me i am	Condition after
physician told me i have	Condition after
physician told me i have a	Condition after
physician said i m	Condition after
physician said i am	Condition after
physician said i have	Condition after
physician said i have a	Condition after
dr diagnosed me with	Condition after
dr diagnosed me as	Condition after
doctor diagnosed me with	Condition after
doctor diagnosed me as	Condition after
physician diagnosed me with	Condition after
physician diagnosed me as	Condition after
had a	Condition after
just had a	Condition after
just got a	Condition after
recently got a	Condition after
i had a	Condition after
i have	Condition after
i have the	Condition after
i have a	Condition after
i got	Condition after
i got the	Condition after
i got a	Condition after
got a	Condition after
have a	Condition after
now i am	Condition after
now i m	Condition after
now i have	Condition after
now i have the	Condition after
now i have a	Condition after

**Pattern (cont.)**	**Relative Condition Position (cont.)**
now i got	Condition after
now i got the	Condition after
now i got a	Condition after
diagnosed	Condition after
why have i got	Condition after
why have i got a	Condition after
why did i get	Condition after
why did i get a	Condition after
why do i have	Condition after
why do i have a	Condition after
why did i have a	Condition after
why was i diagnosed with	Condition after
my	Condition after
diagnosed me with	Condition after
diagnosed me as	Condition after
diagnosed as having	Condition after
diagnosed as having a	Condition after
diagnosed as being	Condition after
